# Temporal trend of the dropout rate and vaccination coverage of the triple viral vaccine in Brazil, 2014-2021

**DOI:** 10.1590/S2237-96222023000300004.EN

**Published:** 2023-10-20

**Authors:** Lívia de Lima Moura, Mercedes Neto, Reinaldo Souza-Santos

**Affiliations:** 1 Fundação Instituto Oswaldo Cruz, Programa de Pós-Graduação em Epidemiologia em Saúde Pública, Rio de Janeiro, RJ, Brazil; 2 Universidade do Estado do Rio de Janeiro, Departamento de Enfermagem de Saúde Pública, Rio de Janeiro, RJ, Brazil; 3 Escola Nacional de Saúde Pública Sergio Arouca, Departamento de Endemias Samuel Pessoa, Rio de Janeiro, RJ, Brazil

**Keywords:** Child Vaccination, Vaccination Coverage, Immunization Schedule, Time Series Studies, Vacunación Infantil, Cobertura de Vacunación, Esquema de Vacunación, Estudios de Series Temporales, Vacinação da Criança, Cobertura Vacinal, Esquema de Vacinação, Estudos de Séries Temporais

## Abstract

**Objective:**

to analyze the temporal trend of coverage and dropout rate for triple viral vaccine in Brazil, according to the country’s Federative Units and Macro-Regions, between 2014 and 2021.

**Methods:**

this was an ecological time series study, using data from the National Immunization Program Information System and the Live Birth Information System; joinpoint regression models were used.

**Results:**

in Brazil as a whole annual vaccination coverage was below 95% and ranged from 92.3% (2015) to 54.4% (2021); the second dose of the vaccine showed a decreasing temporal trend in the period (average change over the period = -5.8; 95%CI -10.5;-0.8); the temporal trends were stationary and decreasing in the country’s Federative Units; the dropout rate ranged from 22.2% (2014) to 37.4% (2021).

**Conclusion:**

there was a downward trend in vaccination coverage and an increase in the dropout rate in Brazil as a whole and in the country’s Federative Units.

## Study contributions

**Main results:**

Annual vaccination coverage was below 95% in Brazil. The second dose of the vaccine showed stationary and decreasing trends in the country’s Federative Units. The dropout rate varied greatly throughout the study period.

**Implications for services:**

The results found regarding the trends serve to inform and point to the urgency of planning actions aimed at improving coverage of the triple viral vaccine nationally in Brazil.

**Perspectives:**

Investments in enhanced training of epidemiological surveillance professionals and enhanced computerized systems are necessary, with a view to continuous monitoring, to support actions to promote better and timely vaccine coverage.

## Resumen

**Objetivo:**

analizar la tendencia temporal de cobertura y tasa deserción de la vacuna triple viral en Brasil, y según Unidades de la Federación y Regiones, entre 2014 y 2021.

**Métodos:**

estudio de serie temporal ecológica, sobre datos de los sistemas del Inmunizaciones y Nacido Vivo; se utilizaron modelos de regresión de punto de inflexión.

**Resultados:**

la cobertura anual de vacunación estuvo por debajo del 95% y osciló entre 92,3% (2015) y 54,4% (2021), en Brasil; la segunda dosis mostró una tendencia temporal decreciente en el período (variación promedia en el periodo = -5,8; IC_95%_ -10,5;-0,8); las tendencias temporales fueron estacionarias y decrecientes en las Unidades de la Federación; la tasa deserción de varió del 22,2% (2014) al 37,4% (2021).

**Conclusión:**

hubo una tendencia a la baja en las coberturas de vacunación y un aumento en la tasa de deserción en Brasil y en las Unidades de la Federación.

## Resumo

**Objetivo:**

analisar a tendência temporal da cobertura e da taxa de abandono da vacina tríplice viral no Brasil, nas Unidades da Federação (UFs) e grandes regiões nacionais, entre 2014 e 2021.

**Métodos:**

estudo ecológico de série temporal, sobre dados do Sistema de Informações do Programa Nacional de Imunizações e do Sistema de Informações sobre Nascidos Vivos; foram utilizados modelos de regressão por pontos de inflexão.

**Resultados:**

as coberturas vacinais anuais, no Brasil como um todo, estiveram abaixo de 95%, variando de 92,3% (2015) a 54,4% (2021); a segunda dose da vacina apresentou tendência temporal decrescente no período (variação média no período = -5,8; IC_95%_ -10,5;-0,8); as tendências temporais foram estacionárias e decrescentes nas UFs; a taxa de abandono variou de 22,2% (2014) a 37,4% (2021).

**Conclusão:**

houve tendência de queda da cobertura vacinal e aumento da taxa de abandono, no Brasil como um todo e nas UFs.

## INTRODUCTION

Epidemiological surveillance, when integrated with immunization actions, enables control, eradication and elimination of vaccine-preventable diseases, promoting improvement in the population’s health.^1^ ^),(^ ^2^ However, the benefits of immunization are unequally distributed: among poorer, more marginalized and more vulnerable populations, access to these benefits is limited to immunization services.^3^

The Immunization Agenda 2030 (IA2030) aims to improve the global population’s access to primary health care and achieve universal coverage of vaccine products. In this sense, childhood vaccination is essential for strengthening public health policies, as well as implementation and progress of immunization programs worldwide.^3^

Several countries achieved improvement in child vaccination coverage between 1980 and 2010.^4^ However, in the 2010s, with the introduction and expansion of new vaccines, particularly in Latin America and the Caribbean, reductions in vaccination coverage were seen, with fewer countries in these regions of the Americas achieving 90% coverage for five of the nine childhood vaccines between 2013 and 2017: only 61% of Latin American and Caribbean countries achieved 90% coverage for the first dose of the triple viral vaccine in 2017.^4^ ^),(^ ^5^

Difficulties in achieving or maintaining the immunization coverage target are recurrent. In 2020 especially, during the early stages of the novel coronavirus (COVID-19) pandemic, routine childhood immunization services were interrupted due to social distancing measures taken with the aim of preventing SARS-CoV-2 transmission. Consequently, mass vaccination campaigns intended to prevent diseases such as measles, meningitis and polio were not undertaken.^6^

The Brazilian National Immunization Program has achieved worldwide recognition, given the geographic dimension and complexity of operations involved in vaccination campaigns, routine vaccination and vaccine blockades in the country.^7^ ^),(^ ^8^

The National Immunization Program offers, free of charge, a variety of immunobiologics for different age groups, from childhood to old age. Through the population’s adherence to vaccination and timely health surveillance, measles transmission in the Americas was interrupted.^9^ ^),(^ ^10^ Measles is an extremely contagious disease, it can cause serious complications and even death, especially in children under 5 years of age and malnourished children.^11^ However, the circulation of measles in other regions of the world led to the reintroduction of the virus in Brazil in 2018,^12^ associated with the drop in vaccination coverage in the country.^10^ ^),(^ ^13^ ^),(^ ^14^

Vaccination coverage is one of the indicators capable of evaluating the performance of vaccination strategies, when measuring the effect of the intervention on an eligible population. Another indicator of vaccination coverage is the dropout rate, which estimates the population’s adherence to the vaccination schedule proposed by the Brazilian National Immunization Program, that is, how many people started but did not complete the vaccination schedule. Vaccination coverage also estimates the effectiveness of interventions, compared to programmed actions.^15^ ^),(^ ^16^

Surveillance of immunization indicators is essential for achieving and maintaining established coverage targets, aiming to protect the population from vaccine-preventable diseases, especially those that affect children.^15^ ^),(^ ^17^

Brazil offers triple viral vaccination - against measles, mumps and rubella (MMR) - on the childhood vaccination schedule, with a first dose at 12 months old; and the second dose of MMR vaccine or, alternatively, a dose of tetraviral vaccine - against measles, mumps, rubella and varicella (MMRV) at 15 months old. This has been the National Immunization Program guideline since 2014.

The objective of this study was to analyze the temporal trend of MMR vaccination coverage and dropout rate in Brazil, according to the country’s Federative Units (FUs) and Macro-Regions, from 2014 to 2021.

## METHODS

This was an ecological time series study, using data from the National Immunization Program Information System (*Sistema de Informações do Programa Nacional de Imunizações* - SI-PNI)^18^ and the Live Birth Information System (*Sistema de Informações sobre Nascidos Vivos* - SINASC),^19^ for the period 2014-2021, taking the Brazilian territory as a whole, its FUs and its macro-regions as units of analysis.

The SI-PNI system aggregates information related to the records of administered vaccine doses, by period of time and geographic area of vaccine administration.^18^ The SINASC system holds information regarding births registered in Brazil.^19^ The two databases are freely accessible, being made available by the Brazilian National Health System Department of Information Technology (*Departamento de Informática do Sistema Único de Saúde* - DATASUS).^18^ ^),(^ ^19^

We consulted the SI-PNI and SINASC records and processed the resulting data using the Health Information Tabulator (*Tabulador de Informações em Saúde* - TabNet), an application made available by DATASUS.^18^ ^),(^ ^19^ Data from both systems were accessed on October 25, 2022 and filtered using TabNet, as follows:

a) Administered doses - SI-PNI

- period (2014 - 2021);

- FU;

- imunobiolologic (MMR and MMRV vaccines); and

- dose (1^st^ dose and 2^nd^ dose);

b) Live birth population - SINASC

- period (2014 - 2021);

- FU;

- year of birth; and

- birth according to mother’s place of residence.

Vaccination coverage was calculated using the following formula:

First dose vaccination coverage %=number of doses administered to children aged 12 months oldlive birth populationx100

Second dose vaccination coverage %=number of doses administered to children aged 15 months oldlive birth populationx100

When selecting the “second dose” variable, we compared the amounts of MMR vaccine and MMRV vaccine administered in each FU and opted for the vaccine with the highest number of doses administered there. This procedure was necessary as there was variation in the distribution logistics of these vaccines in the Brazil throughout the analyzed period.^20^ The median value was used when calculating vaccine coverage by macro-region.

The dropout rate was calculated based on the first administered doses of the MMR vaccine and the second administered doses of the MMR or MMRV vaccines, using the same choice criterion defined for calculating vaccination coverage. The dropout rate was calculated using the following formula:

Dropout rate %=(number of first doses administered- number of second doses administerednumber of first doses administeredx100

When FUs had inconsistent dropout rates, such as values < 1% or negative values, these were replaced by the dropout rate value for the previous year.

Joinpoint regression analysis models based on the Monte Carlo permutation method were used for temporal analysis of vaccination coverage and dropout rate. This regression model verifies whether a line with multiple points is statistically better for describing the temporal evolution of vaccination coverage and dropout rate, compared to a straight line. Classifying temporal trend as not significant (p-value > 0.05), positive (p-value < 0.05 and positive regression coefficient) or negative (p-value < 0.05 and negative regression coefficient) allowed us to calculate annual percentage change (APC) and the average change over the period (ACP). In the regression model, years with a dropout rate < 1 were excluded, by FU.^21^ ^),(^ ^22^ ^)^ A 95% confidence interval (95%CI) was used for all temporal trends.

We generated thematic maps of vaccination coverage ACP and dropout rate ACP per FU. The ACP value strata used in the thematic maps were obtained by adopting the QGIS program natural breaks procedure.

The digital grid for Brazil and its Federative Units was obtained from the webpage of the Brazilian Institute of Geography and Statistics (*Instituto Brasileiro de Geografia e Estatística* - IBGE) (https://www.ibge.gov.br/geociencias/downloads-geociencias.html), which we accessed on December 29, 2022.

In order to perform the analyses, we used the Join Point Regression Program, version 4.9.1.0, dated April 2022 (Statistical Research and Applications Branch, National Cancer Institute), and the QGIS Geographic Information System.^23^

As only secondary public domain and freely accessible data sources were used, the study project did not need to be submitted to a Research Ethics Committee.

## RESULTS

**Figure 1:**
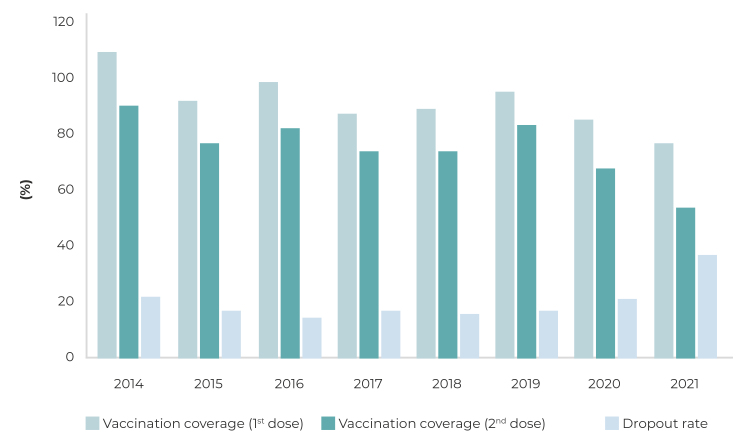
Vaccination coverage (1^st^ dose and 2^nd^ dose) and dropout rate, Brazil, 2014-2021

In Brazil, MMR vaccine coverage ranged from 92.3% to 54.4% between 2015, 2017, 2018, 2020 and 2021. As for the second dose, MMR or MMRV vaccine coverage was below 95% in the period studied period. The dropout rate remained high throughout the period, ranging from 22.2% (2014) to 37.4% (2021) (Figure 1).

For Brazil as a whole, the temporal trend as per the MMR vaccine first dose coverage regression model was not significant, both in the first period, from 2014 to 2019 (APC = -2.4; 95%CI -8.6;4.2), and in the second period, from 2019 to 2021 (APC = -6.7; 95%CI -30.3;25.1) (Table 1).

**Table 1:** Temporal trends of MMR (1^st^ dose), and MMR or MMRV (2^nd^ dose) vaccination coverage, according to joinpoint regression, in the national macro-regions and Federative Units, Brazil, 2014-2021

Region/ Federative Unit	1^st^ dose			2^nd^ dose		
	Period	APC^a^ (95%CI^b^)	Trend^c^	Period	APC^a^ (95%CI^b^)	Trend^c^
North	2014-2019	-4.3 (-8.6;0.1)	Not significant	2014-2019	-1.1 (-10.5;9.3)	Not significant
	2019-2021	-7.9 (-24.8;12.7)		2019-2021	-23.2 (-50.8;20.0)	
RO	2014-2016	-9.7 (-36.0;27.4)	Not significant	2014-2019	-5.0 (-11.1;1.5)	Not significant
	2016-2021	-3.9 (-11.0;3.8)		2019-2021	-23.5 (-43.1;2.9)	
AC	2014-2019	-2.5 (-6.8;2.0)	Not significant	2014-2019	6.0 (-6.0;19.5)	Not significant
	2019-2021	-11.2 (-27.4;8.7)		2019-2021	-38.9 (-64.3;4.5)	
AM	2014-2016	-9.0 (-37.0;31.5)	Not significant	2014-2019	-0.9 (-10.2;9.4)	Not significant
	2016-2021	-2.4 (-10.1;6.0)		2019-2021	-23.5 (-50.8;18.8)	
RR	2014-2019	-9.4^c^ (-13.4;-5.1)	Negative	2014-2019	-4.0^c^ (-7.6;-0.3)	
	2019-2021	-0.3 (-18.8;22.5)	Not significant	2019-2021	-27.5^c^ (-38.7;-14.2)	Negative
PA	2014-2016	-16.0 (-49.0;38.4)	Not significant	2014-2019	5.4 (-11.5;25.5)	Not significant
	2016-2021	-0.6 (-11.1;11.2)		2019-2021	-32.4 (-69.1;47.8)	
AP	2014-2019	-5.9 (-15.1;4.3)	Not significant	2014-2019	-4.2 (-16.6;10.0)	Not significant
	2019-2021	-9.9 (-43.0;42.5)		2019-2021	-28.6 (-61.6;32.6)	
TO	2014-2017	-4.9 (-17.2;9.2)	Not significant	2014-2019	1.7 (-16.9;24.4)	Not significant
	2017-2021	-0.4 (-8.8;8.6)		2019-2021	-20.1 (-67.6;97.0)	
Northeast	2014-2019	-2.5 (11.2;7.1)	Not significant	2014-2019	-3.4 (-9.8;3.5)	Not significant
	2019-2021	-9.1 (-40.2;38.2)		2019-2021	-17.9 (-39.6;11.5)	
MA	2014-2016	-15.1 (-43.8;28.2)	Not significant	2014-2016	-18.8 (-59.2;61.7)	Not significant
	2016-2021	-3.6 (-12.1;5.7)		2016-2021	-4.2 (-17.9;11.8)	
PI	2014-2016	-4.9 (-31.6;32.0)	Not significant	2014-2019	0.6 (-9.4;11.7)	Not significant
	2016-2021	0.7 (-6.4;8.4)		2019-2021	-14.7 (-46.6;36.3)	
CE	2014-2019	-4.2 (-14.5;7.3)	Not significant	2014-2019	-5.7 (-16.3;6.2)	Not significant
	2019-2021	-11.1 (-46.5;47.9)		2019-2021	-14.3 (-49.6;45.7)	
RN	2014-2019	-2.4 (-11.4;7.5)	Not significant	2014-2016	-17.4 (-71.0;135.1)	Not significant
	2019-2021	-8.3 (-40.4;41.3)		2016-2021	-2.6 (-22.9;23.0)	
PB	2014-2019	-2.0 (-10.9;7.9)	Not significant	2014-2019	1.0 (-11.7;15.5)	Not significant
	2019-2021	-12.7 (-43.1;33.9)		2019-2021	-23.3 (-57.9;39.7)	
PE	2014-2019	-1.0 (-11.9;11.2)	Not significant	2014-2019	-3.9 (-4.5;-3.3)	Not significant
	2019-2021	-15.7 (-49.9;41.9)		2019-2021	-20.5 (-22.8;-18.1)	
AL	2014-2019	-1.5 (-7.2;4.5)	Not significant	2014-2019	-4.8 (-10.4;1.2)	Not significant
	2019-2021	-12.9 (-33.1;13.4)		2019-2021	-14.9 (-35.1;11.5)	
SE	2014-2019	-0.7 (-6.4;5.4)	Not significant	2014-2019	-2.5 (-6.2;1.4)	Not significant
	2019-2021	-7.8 (-29.1;20.1)		2019-2021	-7.6 (-22.3;10.0)	
BA	2014-2016	-13.5 (-45.8;38.0)	Not significant	2014-2016	-16.1 (-58.5;69.6)	Not significant
	2016-2021	-2.0 (-11.7;8.8)		2016-2021	-3.8 (-17.8;12.6)	
Southeast	2014-2019	-1.5 (-8.1;5.6)	Not significant	2014-2019	-1.5 (-4.4;1.5)	Not significant
	2019-2021	5.6 (-30.8;28.7)		2019-2021	-9.0 (-20.4;4.1)	
MG	2014-2019	-1.2 (-6.2;4.1)	Not significant	2014-2019	1.8 (-11.2;16.8)	Not significant
	2019-2021	-4.3 (-24.3;20.8)		2019-2021	-11.1 (-51.9;64.2)	
ES	2014-2019	-1.7 (-11.2;8.8)	Not significant	2014-2019	-1.8 (-8.0;4.9)	Not significant
	2019-2021	-4.6 (-39.4;50.3)		2019-2021	-7.8 (-31.2;23.5)	
RJ	2014-2019	-1.8 (-9.1;6.2)	Not significant	2014-2019	-4.5 (-6.8;-2.2)	Not significant
	2019-2021	-23.7 (-46.2;8.1)		2019-2021	-27.8 (-35.2;-19.6)	
SP	2014-2019	-1.1 (-4.5;2.5)	Not significant	2014-2019	-2.4 (-5.0;0.2)	Not significant
	2019-2021	-4.3 (-18.4;12.4)		2019-2021	-9.4 (-19.7;2.3)	
South	2014-2017	-5.1 (-14.2;4.9)	Not significant	2014-2019	1.2 (-9.8;13.5)	Not significant
	2017-2021	0.6 (-5.6;7.2)		2019-2021	-9.6 (-46.0;51.2)	
PR	2014-2017	-5.6 (-13.1;2.5)	Not significant	2014-2019	1.7 (-7.9;12.3)	Not significant
	2017-2021	0.9 (-4.2;6.3)		2019-2021	-11.1 (-42.9;38.5)	
SC	2014-2017	-5.3 (-15.8;6.4)	Not significant	2014-2019	0.9 (-9.8;12.9)	Not significant
	2017-2021	0.0 (-7.1;7.7)		2019-2021	-9.3 (-45.1;49.8)	
RS	2014-2016	-5.7 (-32.9;32.6)	Not significant	2014-2019	3.4 (-10.0;18.8)	Not significant
	2016-2021	0.2 (-7.1;8.2)		2019-2021	-17.9 (-55.9;53.1)	
Midwest	2014-2017	-7.0 (-23.3;12.7)	Not significant	2014-2019	0.2 (-14.0;16.6)	Not significant
	2017-2021	- 0.5 (-11.9;12.3)		2019-2021	-23.2 (-61.1;51.7)	
MS	2014-2016	-12.6 (-47.1;44.5)	Not significant	2014-2019	-0.2 (-17.1;20.1)	Not significant
	2016-2021	-3.0 (-13.3;8.6)		2019-2021	-32.8 (-70.7;54.1)	
MT	2014-2017	-7.1 (-20.7; 8.9)	Not significant	2014-2019	-1.5 (-11.9;10.1)	Not significant
	2017-2021	-1.0 (-10.4;9.5)		2019-2021	-23.2 (-53.4;26.6)	
GO	2014-2016	-11.5 (-28.2;9.1)	Not significant	2014-2019	0.5 (-13.6;17.0)	Not significant
	2016-2021	-0.7 (-5.3;4.0)		2019-2021	-18.1 (-58.4;61.1)	
DF	2014-2016	5.2 (-12.3;26.3)	Not significant	2014-2016	14.0 (-41.9;123.8)	Not significant
	2016-2021	-1.9 (-5.9;2.2)		2016-2021	-8.9 (-21.6;6.0)	
Brazil	2014-2019	-2.4 (-8.6;4.2)	Not significant	2014-2019	-1.6 (-7.0;4.2)	Not significant
	2019-2021	-6.7 (-30.3;25.1)		2019-2021	-15.4 (-34.4;9.0)	

a) APC: Annual percentage change; b) 95%CI: 95% confidence interval; c) significance teste using the Monte Carlo permution method.

As for coverage of the second MMR dose or its replacement by a dose of MMRV vaccine, the regression model showed the same periods of non-significant temporal vaccine coverage trends as the first dose. However, for the period as a whole, from 2014 to 2021, a negative trend was found (ACP = -5.8; 95%CI = -10.5;-0.8), from 91.0% (2014) to 54.4% (2021) (Figure 1).

The temporal trend in the dropout rate regression model was not considered to be significant, both in the period 2014-2019 and also in the period 2019-2021.

*Brazilian macro-regions*

The results of the temporal trend analysis of MMR vaccine first dose coverage for the Brazilian macro-regions were not significant for the North, Northeast and Southeast regions, from 2014 to 2019 (Table 1).

**Figure 2:**
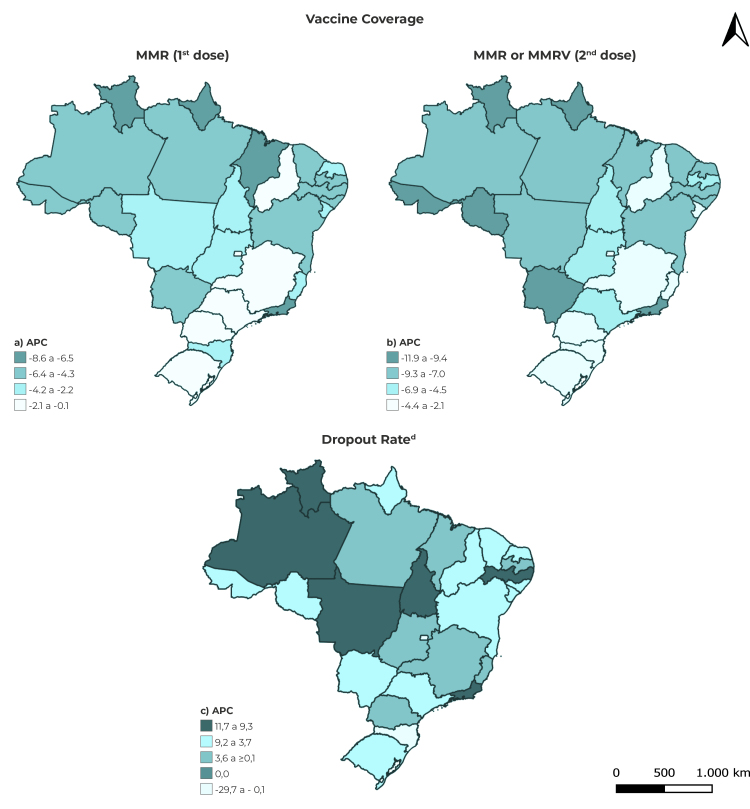
Spatial distribution of annual average percentage changes in immunization indicators and classification of the dropout rate trend in the Federative Units, Brazil, 2014-2021

The North and Northeast regions showed the same temporal behaviors as Brazil as a whole for first dose MMR vaccine coverage, between 2014 and 2021. The trend was negative (ACP = -5.4; 95%CI -9.2;-1.4), decreasing from 105.0% (2014) to 71.0% (2021) in the Northern region (Table 2 and Figure 2A). In the case of MMR (or MMRV) vaccine second dose coverage, a non-significant temporal trend prevailed in the Brazilian regions. A negative trend was found for the Southeast region (ACP = -3.7; 95%CI -6.3;-1.1), falling from 92% (2014) to 66.6% (2021) (Table 2 and Figure 2B). The dropout rate was not significant in any of the Brazilian regions throughout the study period (Table 3).

**Table 2:** MMR (1^st^ dose), and MMR or MMRV (2^nd^ dose), in the national macro-regions and Federative Units, Brazil, 2014-2021

Region/ Federative Unit	1^st^ dose				2^nd^ dose			
	Period	Population < 1 year	Doses administered	Coverage (%)	Period	Population < 1 year	Doses administered	Coverage (%)
North	2014-2019	316,408	277,688	87.7	2014-2019	316,408	213,955	67.6
	2019-2021	305,655	234,748	76.8	2019-2021	305,655	173,553	56.7
RO	2014-2016	27,739	34,208	123.3	2014-2019	27,534	24,929	90.5
	2016-2021	26,803	26,703	99.6	2019-2021	26,208	17,165	65.4
AC	2014-2019	16,559	13,941	84.1	2014-2019	16,559	10,089	60.9
	2019-2021	15,521	11,294	72.7	2019-2021	15,521	7,955	51.2
AM	2014-2016	80,621	81,868	101.5	2014-2019	78,819	59,222	75.1
	2016-2021	76,958	64,709	84.0	2019-2021	76,297	46,129	60.4
RR	2014-2019	11,798	10,820	91.7	2014-2019	11,798	9,811	83.1
	2019-2021	14,047	9,133	65.0	2019-2021	14,047	7,627	54.2
PA	2014-2016	143,580	129,723	90.3	2014-2019	141,068	79,391	56.2
	2016-2021	137,067	98,221	71.6	2019-2021	134,739	70,573	52.3
AP	2014-2019	15,761	14,049	89.1	2014-2019	15,761	11,598	73.5
	2019-2021	14,874	10,416	70.0	2019-2021	14,874	7,472	50.2
TO	2014-2017	24,641	23,828	96.7	2014-2019	24,641	19,113	77.5
	2017-2021	24,464	21,041	86.0	2019-2021	24,464	17,422	71.2
Northeast	2014-2019	825,948	808,278	97.8	2014-2019	825,948	605,616	73.3
	2019-2021	782,217	656,844	83.9	2019-2021	782,217	480,680	61.4
MA	2014-2016	117,317	123,694	105.4	2014-2016	117,317	92,323	78.6
	2016-2021	111,018	82,263	74.0	2016-2021	111,018	59,086	53.2
PI	2014-2016	48,597	41,195	84.7	2014-2019	48,444	31,034	64.0
	2016-2021	47,236	38,943	82.4	2019-2021	46,130	27,876	60.4
CE	2014-2019	129,346	148,190	114.5	2014-2019	129,346	118,188	91.3
	2019-2021	124,331	113,893	91.6	2019-2021	124,331	90,104	72.4
RN	2014-2019	47,381	43,787	92.4	2014-2016	48,605	38,896	80.0
	2019-2021	43,697	37,013	84.7	2016-2021	45,131	26,386	58.4
PB	2014-2019	58,081	56,730	97.6	2014-2019	58,081	39,092	67.3
	2019-2021	56,819	48,964	86.1	2019-2021	56,819	35,275	62.0
PE	2014-2019	138,699	145,678	105.0	2014-2019	138,699	105,147	75.8
	2019-2021	130,107	112,742	86.6	2019-2021	130,107	80,129	61.5
AL	2014-2019	51,028	53,423	104.6	2014-2019	51,028	37,963	74.3
	2019-2021	48,828	43,961	89.4	2019-2021	48,828	29,147	59.6
SE	2014-2019	33,925	30,955	91.24	2014-2019	33,925	25,193	76.5
	2019-2021	32,088	27,006	84.1	2019-2021	32,088	21,777	67.8
BA	2014-2016	205,344	212,162	103.3	2014-2016	205,344	172,157	83.8
	2016-2021	197,404	160,343	81.2	2016-2021	197,404	119,084	60.3
Southeast	2014-2019	1,161,104	1,131,875	97.4	2014-2019	1,161,104	944,673	81.3
	2019-2021	1,069,265	955,463	89.3	2019-2021	1,069,265	783,218	73.2
MG	2014-2019	262,710	258,335	98.3	2014-2019	262,710	209,693	79.8
	2019-2021	250,429	234,775	93.7	2019-2021	250,429	198,811	79.3
ES	2014-2019	55,893	53,727	96.1	2014-2019	55,893	44,143	78.9
	2019-2021	54,153	48,820	90.1	2019-2021	54,153	41,405	76.4
RJ	2014-2019	226,679	236,052	104.1	2014-2019	226,679	174,434	76.9
	2019-2021	202,079	156,917	77.6	2019-2021	202,079	112,494	55.6
SP	2014-2019	615,819	583,761	94.7	2014-2019	615,819	516,401	83.8
	2019-2021	562,603	514,951	91.5	2019-2021	562,603	430,507	76.5
South	2014-2017	398,260	387,959	97.4	2014-2019	397,648	327,646	82.3
	2017-2021	385,891	346,834	89.8	2019-2021	378,665	308,262	81.4
PR	2014-2017	158,642	158,753	100.0	2014-2019	157,966	134,750	85.3
	2017-2021	151,990	138,624	91.2	2019-2021	148,683	124,132	83.4
SC	2014-2017	95,256	94,730	99.4	2014-2019	96,742	79,433	82.1
	2017-2021	98,361	87,673	89.1	2019-2021	97,954	81,162	82.8
RS	2014-2016	145,837	136,896	93.8	2014-2019	142,940	113,462	79.3
	2016-2021	136,517	122,054	89.4	2019-2021	132,026	102,967	77.9
Midwest	2014-2017	242,517	248,493	102.4	2014-2019	243,529	197,818	81.2
	2017-2021	238,425	205,532	86.2	2019-2021	234,009	160,762	68.6
MS	2014-2016	44,100	54,160	122.8	2014-2019	43,930	39,112	89.0
	2016-2021	42,960	48,429	112.7	2019-2021	42,103	28,936	68.7
MT	2014-2017	55,567	56,248	101.2	2014-2019	56,524	44,894	79.4
	2017-2021	57,769	48,429	83.8	2019-2021	57,642	37,442	64.9
GO	2014-2016	100,235	101,854	101.6	2014-2019	98,485	73,892	75.0
	2016-2021	95,600	81,222	84.9	2019-2021	93,882	63,809	67.9
DF	2014-2016	45,421	37,856	83.3	2014-2016	45,421	34,629	76.2
	2016-2021	42,207	40,226	95.3	2016-2021	45,207	37,010	81.8

*Federative Units*

The results of the temporal trend analysis of MMR vaccine first dose coverage by FU showed a negative trend in Roraima (APC = -9.4; 95%CI -13.4;-5.1), from 105.0% (2014) to 65.1% (2019) (Tables 1 and 2). With regard to the temporal trend in the period as a whole, from 2014 to 2021, Acre and Rio de Janeiro reported negative trends (Figure 2A).

The FUs that make up the Southeast region showed the same temporal behaviors for MMR vaccine first dose coverage as the region as a whole (Table 1).

Regarding MMR vaccine (or MMRV vaccine) second dose coverage, Roraima showed a negative trend in the period 2014-2019 (APC = -4.0; 95%CI -7.6;-0.3) and in the period 2019-2021 (APC = -27.5; 95%CI -38.7;-14.2), ranging from 85.6% to 37.0% in the period as a whole, from 2014 to 2021. In this longer period, negative trends were also found for Rondônia, Amapá, Pernambuco, Alagoas, Sergipe and Rio de Janeiro (Tables 1 and 2; Figure 2B).

Dropout rate trends over time were not assessed for Roraima and the Federal District because they were less than 1% between 2017 and 2019 (Table 3).

**Table 3:** Dropout rate temporal trend, according to joinpoint regression, in the national macro-regions and Federative Units, Brazil, 2014-2021

Region/ Federative Unit	Period	APC^a^ (95%CI^b^)
North	2014-2016	-35.9 (-86.6;207.0)
	2016-2021	26.8 (-10.7;79.9)
RO	2014-2016	-20.0 (-74.4;150.4)
	2016-2021	23.2 (-4.5;59.0)
AC	2014-2019	-23.2 (-47.3;11.9)
	2019-2021	139.0 (-55.7;1188.5)
AM	2014-2019	-10.8 (-43.0;39.5)
	2019-2021	95.1 (-73.6;1341.8)
RR	2014-2021^c^	9.3 (-24.3;57.8)
PA	2014-2019	-21.5 (-56.0;40.3)
	2019-2021	91.4 (-85.7;2465.8)
AP	2014-2016	-33.8 (-82.2;146.3)
	2016-2021	27.7 (-4.8;71.3)
TO	2014-2019	-16.1 (-67.3;115.4)
	2019-2021	128.5 (-96.6;15419.1)
Northeast	2014-2019	0.0 (-26.2;35.6)
	2019-2021	32.1 (-66.1;414.5)
MA	2014-2019	0.5 (-16.3;20.6)
	2019-2021	11.7 (50.6;152.8)
PI	2014-2016	-3.4 (-16.1;11.2)
	2016-2021	42.1 (42.1;-24.3)
CE	2014-2016	43.9 (43.9;252.2)
	2016-2021	-2.5 (-20.2;19.0)
RN	2014-2016	43.2 (-56.8;374.8)
	2016-2021	-2.3 (-25.3;27.7)
PB	2014-2019	-10.9 (-29.2;12.3)
	2019-2021	40.1 (-50.1;293.4)
PE	2014-2016	46.9 (-60.0;439.8)
	2016-2021	-1.0 (-26.0;32.4)
AL	2014-2016	29.3 (-4.6;75.2)
	2016-2021	1.0 (-5.6;8.2)
SE	2014-2018	8.0 (-14.6;36.6)
	2018-2021	1.7 (32.2;42.5)
BA	2014-2019	-0.7 (-22.2;26.8)
	2019-2021	29.6 (-56.6;286.9)
Sortheast	2014-2019	2.2 (-13.2;20.4)
	2019-2021	21.4 (-41.5;152.0)
MG	2014-2017	-35.5 (-85.8;193.2)
	2017-2021	25.8 (51.7;227.6)
ES	2014-2019	-6.0 (-35.3;36.5)
	2019-2021	27.0 (-76.0;573.4)
RJ	2014-2016	37.8 (-53.6;309.0)
	2016-2021	0.4 (-21.3;28.1)
SP	2014-2017	-18.4 (73.2;148.6)
	2017-2021	31.9 (-34.8;166.8)
Sorth	2014-2018	-23.4 (-62.5;56.5)
	2018-2021	45.7 (-52.9;350.8)
PR	2014-2019	-42.1 (-70.8;14.8)
	2019-2021	261.0 (-83.1;7609.6)
SC	2014-2019	-35.7 (-61.0;6.1)
	2019-2021	93.1 (-79.4;1710.0)
RS	2014-2019	-26.7 (-64.6;51.8)
	2019-2021	173.0 (-89.5;6979.6)
Midwest	2014-2016	-55.3 (-93.9;226.1)
	2016-2021	42.9 (-8.4;122.9)
MS	2014-2018	-27.3 (-74.0;102.7)
	2018-2021	82.3 (-64.0;823.2)
MT	2014-2019	-16.7 (-35.5;7.6)
	2019-2021	132.2 (-26.1;629.7)
GO	2014-2019	-24.3 (-58.8;39.0)
	2019-2021	93.1 (-87.2;2822.5)
DF	2014-2021^c^	29.7 (-26.8;130.1)
Brazil	2014-2019	-3.9 (-15.8;9.7)
	2019-2021	49.2 (-17.5;169.9)

a) Annual percentage change; b) 95%CI: 95% confidence interval; c) Without regression results, due to the low values.

Note: All trends were stationary.

Vaccination coverage and dropout rate indicators, according to the period observed, showed different trends within the same region of the country, showing temporal heterogeneity between the FUs (Tables 1, 2 and 3; Figure 2C).

a), b) and c) ACP: Average change over the period. d) Dropout rate: Rate of vaccinated children that began but did not finish the schedule.

## DISCUSSION

In this study, coverage of both the first MMR vaccine dose and the second MMR dose - or its replacement with a dose of MMRV vaccine - decreased in Brazil as a whole, in the period selected for the study. The FUs, in particular, showed stationary or decreasing trends in vaccine coverage, either for the first or the second vaccine dose, over the period studied.

It should be noted that the temporal trend periods for second dose vaccination coverage were from 2014-2019 and 2019-2021 for all the Brazilian macro-regions. However, some FUs, such as Maranhão, Rio Grande do Norte, Bahia and the Federal District, differ from each other because their trend periods were 2014-2016 and 2016-2020, diverging from the periods applying to their respective macro-regions. These divergences point to the possibility of different factors interfering, at different times, in the vaccination coverage found.^10^

The dropout rate, indicative of the portion of the population that did not complete the vaccination schedule,^15^ had a stationary trend, both in Brazil as a whole and in all the country’s regions. The Northeast and Southeast regions had the same trend periods for the dropout rate (2014-2019 and 2019-2021), in relation to Brazil as a whole. Among the FUs, only Rondônia, Maranhão, Pernambuco, Sergipe, Bahia, São Paulo and Santa Catarina reported the same periods as Brazil and the Northeast and Southeast regions, in the temporal context of the study.

In the FUs with periods and trend behaviors different from their respective regions, heterogeneity can be seen within their regions, with regard to vaccination coverage and dropout rates.

Childhood vaccine coverage has made progress. However, in the period from 2010 to 2019, coverage of the third dose of DTP vaccine (diphtheria, tetanus and pertussis), the first dose of MMR vaccine and third dose of vaccine against poliomyelitis stagnated or decreased. Worldwide, 94 countries and territories (46%) recorded reductions in these coverage levels.

Global coverage of the first MMR vaccine dose stagnated at a level between 84% and 86% in the period 2010-2019, while coverage of the second MMR vaccine dose has increased from 42% to 71%, reflecting the introduction of the second dose in many countries.^24^

The second dose of the MMR and/or MMRV vaccine is not included in all vaccination schedules worldwide.^24^ In the case of Brazil, the inclusion of the second dose occurred in 2013 and its coverage remained below the target recommended by the National Immunization Program (< 95%) between 2014 and 2021.^25^

Heterogeneity of vaccination coverage of nine vaccines on the childhood schedule, among the Brazilian regions, is more prominent in the Midwest, where it was higher (90.6%), compared to the other regions of the country from 2015 to 2019. The FUs that make up the Northern region also showed temporal heterogeneity in the vaccination coverage of nine vaccines on the childhood schedule, with Rondônia standing out with the best coverage (100%) and Pará with the worst coverage (69.4%), also between 2015 and 2019.^26^

A study was conducted in Serbia on the temporal trends of mandatory childhood vaccination coverage between 2000 and 2017, using linear regression and joinpoint statistical methods. The linear regression revealed a significant drop in coverage of the first doses of poliomyelitis, DTP and MMR vaccines.

In the same period, coverage of all subsequent revaccinations decreased significantly.^27^

The impact of the COVID-19 pandemic contributed to an 84% reduction in global coverage of the first MMR vaccine dose, while coverage of the second MMR or MMRV vaccine dose remained stable, with average percentage values of 71% in 2019 and 70% in 2020, estimated based on recurring heterogeneity between the different regions of the world.^28^

High dropout rates are repeatedly found globally: in 2017, 6.2 million (31%) children started but did not complete the DTP vaccine schedule.^29^ It is noteworthy that high dropout rates can mean reduced herd immunity and increased of cases of vaccine-preventable diseases.^15^

The United Nations Development Programme reported that only 1% of the 10.7 billion doses of vaccines distributed worldwide were administered in low-income countries as at mid-2022. As such, the Immunization Agenda 2030 can not only help improve the quality of coverage estimates, but also help to identify and reach people needing to be vaccinated, including those from displaced and marginalized populations who are not being fully immunized in a timely manner.^28^ ^),(^ ^29^

Barriers to vaccine equity may be related to lack of credibility of the information and guidance provided by health authorities and health professionals regarding vaccination. “Fake news” decreases the population’s confidence in the health system and, in particular, with regard to vaccination actions and campaigns. However, as government policies expand the availability of vaccines and health professionals engage in the vaccination process, this process is strengthened, as is the health system as a whole.^10^

As for the limitations of this study, it is worth mentioning possible uncertainties/imprecision in the calculation of vaccination coverage, when the denominator used to calculate the rates includes population estimates that underestimate or overestimate the population under 1 year old, in addition to the insufficient number of observations analyzed. Moreover, constant changes in immunization information systems can lead to typing errors and information that is not migrated from one system to another and, consequently, underestimated vaccine coverage and an overestimated dropout rate.

This study makes progress by identifying temporal heterogeneity and periods of trends, in addition to differences in the geographical distribution of indicators, this being a form of analysis that should be incorporated into the routine of health services, in addition to addressing the dropout rate, which is an immunization indicator little discussed in the scientific literature.

We conclude that further studies are needed to characterize the spatial heterogeneity of MMR vaccine coverage and its dropout rate, as well as possibly associated factors. Furthermore, immunization services need to monitor temporal trends in vaccine coverage, with the aim of intensifying educational actions aimed at greater timely adherence by the population to vaccination.
